# Editorial to “The effect of ablation settings on lesion characteristics with DiamondTemp ablation system: An ex vivo experiment”

**DOI:** 10.1002/joa3.13013

**Published:** 2024-02-29

**Authors:** Piotr Futyma, Shaojie Chen

**Affiliations:** ^1^ St. Joseph's Heart Rhythm Center Rzeszów Poland; ^2^ Medical College University of Rzeszów Rzeszów Poland; ^3^ Cardioangiologisches Centrum Bethanien (CCB), Kardiologie, Medizinische Klinik III, Agaplesion Markus Krankenhaus, Akademisches Lehrkrankenhaus Der Goethe‐Universität Frankfurt Am Main Frankfurt am Main Germany

**Keywords:** ablation catheter, diamond‐tip, high‐power short‐duration ablation, tissue preconditioning, ventricular tachycardia

The history of radiofrequency catheter ablation of atrial fibrillation (AF) is marked by continuous efforts to enhance efficacy and safety. Over the years, studies have emphasized the importance of achieving adequate lesion depth and contiguous lesion formation for successful AF ablation. The introduction of technologies and strategies, such as high‐power short duration (HPSD) ablation, has provided valuable insights into optimizing lesion characteristics, leading to improved performance.^1^, ^2^ High‐power short duration ablation however did not come without risk of overshooting the radiofrequency current using up‐to‐date available equipment.^3^ Thus, new designs of ablation catheters were introduced into practice, equipped with specialized sensitive temperature sensors, capable to track and subsequently manage any possible dangerous overheating during HPSD.^4^

With the widespread use of the systems capable for HPSD, it has become tempting to use these advanced tools also in the ventricles. Cohorts of patients with frequently challenging ventricular tachycardias could possibly also benefit from more rapid and effective lesion formation provided by high‐power techniques. However, heterogeneity and thickness of ventricular tissue would sometimes require extending duration of radiofrequency delivery beyond HPSD protocols. Such extension of high‐power applications would likely increase the likelihood of adverse events.^2^

In the study published in the current issue of *Journal of Arrhythmia*, Nomura et al.^5^ aimed to investigate the performance of ablation catheter, designed specifically for performance at high‐power values. On this occasion, the authors tested ablation catheter's capabilities in the setting of prolonged applications, which are sometimes required in the ventricles, especially in the setting of intramural substrates. The tested catheter is provided with distally positioned external thermocouples, which allow a dedicated generator to rapidly modulate power if needed. This allows the ablation system to safely operate at high‐power values. Additionally, the tip of the ablation catheter is equipped with two industrial‐diamond components which enhance heat transfer. Such features aim to improve overall performance, efficacy, and safety of the modern high‐power approach.

Regarding efficacy, Nomura et al. showed that the diamond‐tip catheter performed better when it was positioned in the perpendicular orientation to the tissue surface. Lesions were deeper, especially when the duration of application was extended above 30 s. Moreover, if the application was repeated, the authors noticed the incremental grow in the lesion size. This fact suggests a role of tissue preconditioning using radiofrequency current: While the initial lesion fails to achieve the maximal lesion depth, it generates lower impedance of the tissue, allowing better penetration during the next radiofrequency application.

Given the fact that the investigated catheter is not equipped with contact sensor, Nomura et al. took their opportunity and, using their original ex‐vivo setup, tested the impact of force applied on lesion's geometry. Further increase in lesion size was observed at elevated contact of 30 g. Steam‐pops were exclusively present during such perpendicular catheter orientation. Interestingly, temperature values calculated from thermistors were significantly lower in perpendicular contact. The authors suggest that such lower temperature levels during applications delivered at “more aggressive” angle can be associated with a unique catheter's design: Both configuration of temperature sensors and location of irrigation holes near the distal thermocouples likely play a role in described discrepancy between lesion depth and temperature rise. The question is, if extended lesions with the catheter combined with temperature differences can bring some risk of char formation at the proximal area of the catheter's tip? Or does the unique dual composite design of the tip transfers the heat rapidly enough to minimize such risk when high‐power applications are prolonged? These aspects appear particularly relevant for those clinicians who aim to facilitate the diamond‐tip catheter for difficult ventricular arrhythmias, which may frequently require extensive lesion set.

The study's results have direct implications for clinicians engaged in catheter ablation procedures. The findings indicate more stable radiofrequency delivery using parallel approach when coupled with precise temperature control and accurate power titration. This allows for extended application durations without inducing adverse effects such as steam pops, while more parallel tip orientation can be helpful for optimizing lesion formation for deep substrates. Future research may benefit from in vivo models and larger cohorts to further validate and extend these results. Clinicians should consider these findings not only when utilizing the currently investigated catheter but also when tackling difficult ventricular arrhythmias with high power using different setups. As technology continues to advance, further research will be crucial for refining high‐power ablation strategies and improving clinical outcomes in the management of cardiac arrhythmias arising from the ventricles.

## CONFLICT OF INTEREST STATEMENT

Dr. Futyma reports patents related with bipolar ablation and high‐voltage ablation and that he has equity in CorSystem. Dr. Chen reports no conflict.
